# Temporal and Spatial Variation (2001–2020) Characteristics of Wind Speed in the Water Erosion Area of the Typical Black Soil Region, Northeast China

**DOI:** 10.3390/ijerph191710473

**Published:** 2022-08-23

**Authors:** Liang Pei, Chunhui Wang, Liying Sun, Lili Wang

**Affiliations:** 1 Key Laboratory of Water Cycle and Related Land Surface Processes, Institute of Geographic Sciences and Natural Resources Research, Chinese Academy of Sciences, Beijing 100101, China; 2 Xinjiang Institute of Ecology and Geography, Chinese Academy of Sciences, Urumqi 830011, China; 3 University of Chinese Academy of Sciences, Beijing 100049, China; 4 State Key Laboratory of Atmospheric Boundary Layer Physics and Atmospheric Chemistry, Institute of Atmospheric Physics, Chinese Academy of Sciences, Beijing 100191, China

**Keywords:** typical black soil region, wind speed, spatial-temporal variation, wind impact, geographic detectors

## Abstract

Soil erosion is one of the driving factors leading to the land degradation in the black soil region of Northeast China. It is of great significance to analyze the temporal and spatial variation characteristics of wind speed there for the study of wind erosion impacts and geomantic erosion. Based on the daily meteorological data of 51 meteorological stations from 2001 to 2020, the interannual variation, seasonal variation, and spatial characteristics of wind speed were analyzed by cumulative anomaly method, Mann–Kendall test method, and Kriging interpolation method. The natural factors affecting wind speed were discussed by using geographic detectors, and the potential effects of wind speed on soil erosion were further analyzed. The results showed that the maximum annual wind speed in the water erosion area of the typical black soil region fluctuated with a decreasing trend. However, the mean annual wind speed demonstrated a decreasing trend before 2014, and then showed increasing trend. The proportion of the meteorological stations with decreasing mean annual wind speed and maximum annual wind speed during years 2001–2020 was 70% and 60%, respectively. The seasonal variation of the mean monthly wind speed and maximum monthly wind speed showed the same trend as Spring > Autumn > Winter > Summer. The spatial variation of the mean annual wind speed and maximum annual wind speed was consistent. According to the results of the geographic detectors, DEM and temperature are the main factors affecting the spatial heterogeneity of the maximum annual wind speed. The area of ‘severe’ and ‘extremely severe’ of wind impacts account for 23.4%, and specific concerns should be paid to the areas of Nenjiang, Yilan, Tonghe, and Baoqing, located in the north and east sides of the study area. The results of the article could provide reference for the study of wind–water complex erosion in the water erosion area of the typical black soil region for better soil erosion control and ecological protection.

## 1. Introduction

There are three large black soil regions in the world, namely, the Great Plains of Ukraine, the Mississippi Plain in the United States, and the Northeast Plain in China [1]. Black soil region has also been severely tested by soil erosion with the processes of development and utilization. In the past, wind erosion was reported as serious in the Great Plains of Ukraine and the Mississippi River Basin of the United States, due to the flat terrain. For example, the hazardous ‘black blizzards’ occurred frequently in these areas in the 1920s and 1930s, due to the excessive cultivation. In the United States, “black blizzards” in 1934 swept away 300 million m^3^ of topsoil and killed livestock along the moving way [2]. In order to protect the black soil from damage, great efforts and huge financial investments were made to investigate the wind erosion and the related conservation tillage practices, such as large-scale construction of farmland protection forests, soil conservation rotation, inter-planting, less tillage and no tillage, and to establish the scientific farming system [3,4]. However, the wind erosion effects on the black soil regions still existed, with great risks to the crop production and ecological quality [5,6,7].

Black soil region is an important commodity grain production based in China [8,9,10]. The outputs of corn and soybean in the black soil region of Northeast China account for 32.8% and 45.8% of the total output of the country, respectively (http://data.stats.gov.cn) (accessed on 5 April 2022). Therefore, the crop production in the black soil region is of significance to food security in China [11,12,13]. At present, the boundary of the black soil region in northeastern China has not been unified yet, mainly due to the different views on the type of soil that ‘black soil’ included [14,15]. Liu et al. tried to delineate the ‘black soil region’ and the ‘typical black soil region’ of northeastern China, indicating that the black soil region mainly includes black soil, chernozem, chestnut soil, gray forest soil, and interspersed meadow soil, and the typical black soil region mainly includes black soil and chernozem [9].

With the development and utilization of black soil, affected by natural and human factors, soil erosion is serious and leads to the severe land degradation in the black soil region of northeastern China, which is especially seriously in the typical black soil region [16,17,18]. As a result, black soil layer thickness is decreasing [19], with soil organic matter losing [19,20] and soil hardening [21,22]. The annual grain yield reduction caused by soil erosion was as high as 14.7%, which seriously affected the sustainable development of agriculture and the national food security of the society [15,23,24].

The water erosion area accounted for more than 19.7% in the black soil region, accounting for 63.7% of the area with soil erosion, which should be specifically concerned (http://www.mwr.gov.cn/) (accessed on 5 April 2022). The previous studies in the typical black soil region mainly focused on the soil erosion under the single erosion force [19,25,26,27], while there were relatively few studies on the complex erosion by multiple forces. Due to its unique geographical environment, the typical black soil region is subject to the complexation erosion of freeze–thaw, wind, and water, which is manifested as the continuity in time and the superposition in space [28]. The complex erosion by wind and water commonly occurs in the typical black soil region. Wind and water jointly or alternatively act on the same cropland, which is different from the erosion process by the single force [29]. In Spring, the wind is strong and the exposed cropland is very vulnerable to wind after harvest, leading to the loss of soil organic matter with the fine particles eroded by wind. In Summer and Autumn, the cropland mainly suffers water erosion due to the high frequency of rainstorms on the cultivated land. Moreover, it was found that wind action leads to the changes of surface soil, which may affect the rainfall infiltration, runoff path, flow velocity, and accelerate water erosion rate in the laboratory [29]. The two erosion processes alternate in time and overlap in space, so the erosion time is prolonged and the degree of soil erosion is intensified [28]. The temporal and spatial variation characteristics of wind force in water erosion areas of the typical black soil region is the basic for understanding the complex erosion by wind and water in the field, which are not clear at present.

In this study, the spatial-temporal variation characteristics of wind speed in recent 20 years were studied based on speed data (2001–2020) in the water erosion area of the typical black soil region, for the basis of understanding the wind impacts on the water erosion and their complex erosion processes. Specifically, the multi-time scale variations and spatial distribution characteristics of the mean wind speed, maximum wind speed, and strong wind days in water erosion areas of the typical black soil region were explored. The driven factors were determined and the degrees of the wind impacts were classified. The study results have laid a foundation for the study on the protection and efficient utilization of black soil resources in the world. It provides a theoretical and scientific basis for the coordinated development of black soil resources, agricultural sustainable development, and food security in northeastern China.

## 2. Materials and Methods

### 2.1. Study Area

The area of the typical black soil region (118–135° E, 44–51° N) was 333,000 km^2^, accounting for 23.0% of the total area of Northeast China [9], spanning about 6 latitudes from north to south and about 17 longitudes from east to west, and is located in the temperate continental monsoon climate region. Overall, 70–80% of the annual rainfall is concentrated in the months of June to September. The annual average rainfall is 400–1200 mm, with serious water erosion.

**Figure 1:**
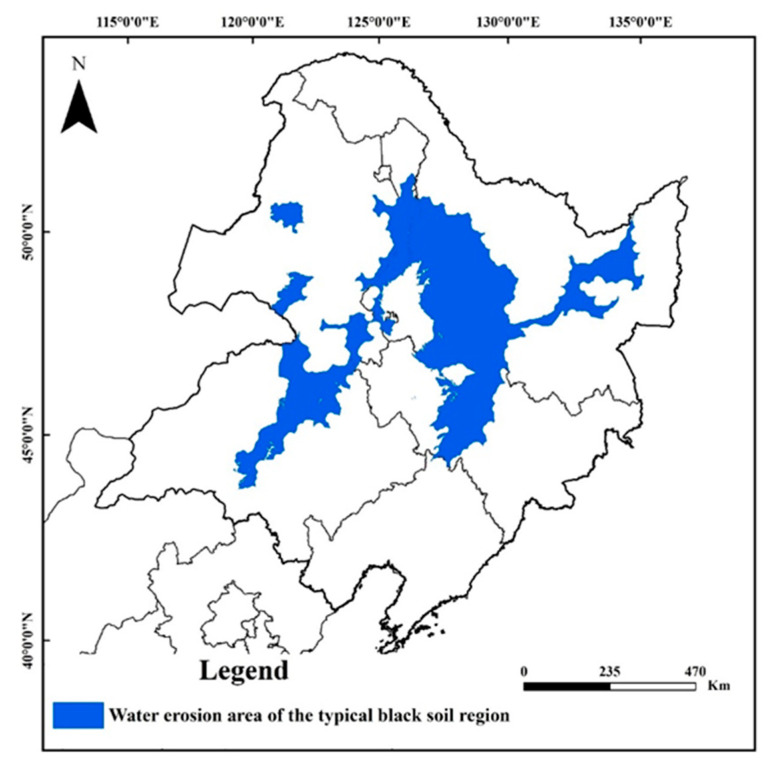
Location of the study area (water erosion area of the typical black soil region).

In this study, the selected study area is the water erosion area in the typical black soil region (Figure 1; 119–135° E, 44–51° N). The water erosion area of typical black soil region was obtained by using spatial superposition technology of ArcGIS software according to the spatial superposition results of typical black soil region and water erosion area, involving 98 counties (cities) in Heilongjiang, Jilin, and Inner Mongolia. The boundary of the water erosion area in China was obtained from the Resource and environment science and data center, Chinese Academy of Sciences (https://www.resdc.cn) (accessed on 8 April 2022), and the typical black soil area was obtained from Global change science research data publishing system (http://www.geodoi.ac.cn) (accessed on 8 April 2022). The main soil types in the study area are black soil, chernozem soil, and interspersed meadow soil.

### 2.2. Data Sources

**Figure 2:**
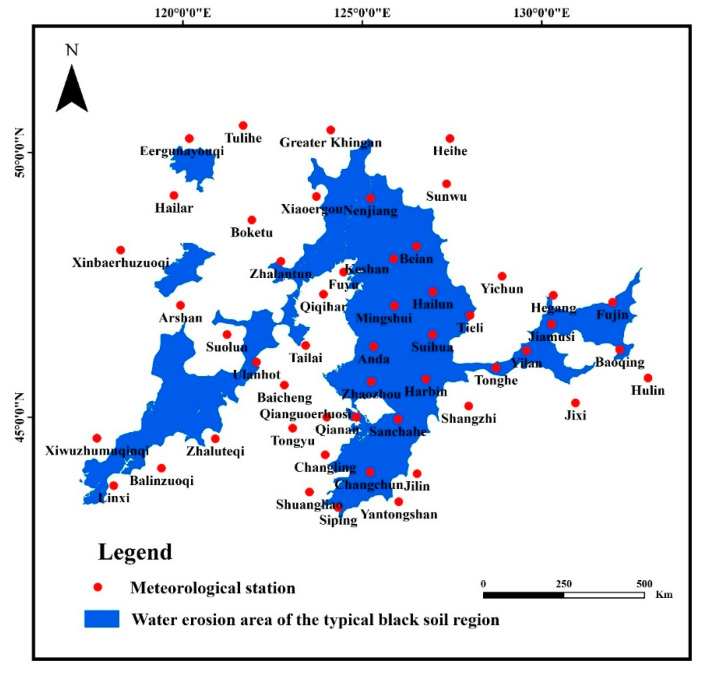
Location of the selected meteorological stations.

The wind speed data was obtained from the China Meteorological Administration, including the daily measured wind speed data of 51 meteorological stations (Figure 2) during the years of 2001–2020. The wind speed indicators include mean wind speed, maximum wind speed, and strong wind days. The four seasons are classified as Spring (March to May), Summer (June to August), Autumn (September to November), and Winter (December, January, and February). Factors of Terrain (Digital Elevation Model (DEM), slope gradient, slope aspect) were obtained from Geospatial Data Cloud (https://www.gscloud.cn/) (accessed on 8 April 2022), factors of geomorphology type (plain, rolling hills, hill, alluvial terrace), vegetation, and climate were obtained from National Catalogue Service For Geographic Information (https://www.webmap.cn/) (accessed on 8 April 2022), and factor of land use type was obtained from GLOBELAND30 (http://www.globallandcover.com/) (accessed on 8 April 2022).

### 2.3. Wind Speed Classification

The mean annual wind speed refers to the average value of wind speed in each year during years of 2001–2020. The mean monthly wind speed refers to the average value of wind speed for each month from 2001 to 2020. The maximum annual wind speed refers to the maximum wind speed in each year from 2001 to 2020. The maximum monthly wind speed refers to the maximum wind speed for each month from 2001 to 2020. A previous study indicated that the threshold wind speed for sand movement in the typical black soil region was 8 m s^−1^, and the soil water content was less than 1.4 g/kg [22,29]. The strong wind days were the average number of days with wind speed greater than 8 m s^−1^ during years of 2001–2020.

### 2.4. Analysis Method

#### 2.4.1. Cumulative Anomaly Method and Mann–Kendall Test Method

The cumulative anomaly method can directly reflect the changing stages of the interannual variation of wind speed [30,31], calculated as:

(1) $$LP_{i}=\sum_{i=1}^{n}\left(R_{i}-\bar{R}\right)$$

where $LP_{i}$ is the cumulative anomaly value in the *i*-th year, *R_i_* is the wind speed in the *i*-th year, and $\bar{R}$ is the mean annual value of the wind speed series. The cumulative anomaly method was used for the stage classifications of wind speed based on the abrupt point in a certain period. The core of this method is that when the measured data is greater than the average value, the cumulative anomaly value increases and the curve shows an upward trend. Otherwise, it shows a downward trend. According to the fluctuation of the curve, the time of abrupt change could be judged, and then different time phases could be divided.

The Mann–Kendall test is a nonparametric statistical test method which can test the variation trend of the time series of variables [32,33,34]. Assuming that *x*_1_, *x*_2_ … *x_n_* are time series variables and *n* is the length of time series, the Mann–Kendall test method defines the statistic *S*:

(2) $$S=\sum_{j=1}^{n-1}\sum_{k=j+1}^{n}sgn\left(x_{k}-x_{j}\right)$$

(3) $$sgn\left(x_{k}-x_{j}\right)=\{\begin{array}{c} 1\;\;\;x_{k}-x_{j}>0\\ 0\;\;\;x_{k}-x_{j}=0\\ -1\;\;x_{k}-x_{j}<0 \end{array}$$

(4) $$Var\left(S\right)=\frac{n\left(n-1\right)\left(2n+5\right)-\sum_{j=1}^{n}t_{j}(t_{j}-1)-\left(2t_{i}+5\right)}{18}$$

(5) $$Z=\{\begin{array}{c} \frac{S-1}{\sqrt{Var\left(S\right)}},\;\;\;\;\;\;\;S>0\\ \;\;\;\;\;\;\;0\;\;\;\;,\;\;\;\;\;\;\;\;S=0\\ \frac{S+1}{\sqrt{Var\left(S\right)}},\;\;\;\;\;S<0 \end{array}$$

where, $x_{j}$, $x_{k}$ is the corresponding measured value of the year. *Var*(*s*) is the variance of the sample. Additionally, $t_{j}$ represents the width of the knot (umber of data with identical VC values in group *j*).

At a given *α* at the confidence level (*α* = 0.05), if │*Z*│ > 1.96, then reject the original assumption. At the confidence level of *α*, there is an obvious upward or downward trend in the time series data. The magnitude of the change trend is express by *β*. The calculation is as follows:

(6) $$\beta =Median\left(\frac{x_{k}-x_{j}}{k-j}\right)\forall j<k$$

If *β* > 0, this indicates an upward trend. If *β* < 0, this indicates a downward trend. In this study, the Mann–Kendall test was used to test the presence of a monotonic increasing or decreasing trend. This method was used to examine the wind speed trend of the meteorological stations for 20 years.

#### 2.4.2. Kriging Interpolation Method

Kriging interpolation is a widely used interpolation algorithm, which interprets the spatial correlation of surface changes by assuming the direction or distance between sampling points [35,36,37,38,39]. The Kriging interpolation method includes data exploration and statistics stage, variation function modeling, and creates surface stage and variance surface stage. The least square algorithm is used to achieve the goal of zero error expectation and minimum variance. Its common formula consists of the weighted sum of data:

(7) $$\hat{Z}\left(x_{0}\right)=\sum_{i=1}^{N}\lambda_{i}Z\left(x_{i}\right)$$

where $Z\left(x_{i}\right)$ is the measured value at the *i*-th position, $\lambda_{i}$ is the unknown weight of the measured value at the *i*-th position, $x_{0}$ is the predicted position, and *N* is the total number of measured values. The Kriging interpolation method was used to predict and analyze the spatial variation of the wind speed indicators.

#### 2.4.3. Geographic Detectors

The geographic detectors method was proposed by Wang Jinfeng et al. [40], which is used to detect and reveal the driving factors of the spatial diversity using statistical method. Geographic detectors include four sub-detectors: Factor detector, Interaction detector, Ecological detector, and Risk detector. Factor detector could determine the influencing degree of detection factors on the spatial heterogeneity of mean annual wind speed and maximum annual wind speed, which is expressed by *q* value. The larger the *q* value, the greater degree of influencing factor on wind speed.

(8) $$q=1-\frac{\sum_{h=1}^{L}N_{h}\sigma_{h}^{2}}{N\sigma^{2}}$$

where *q* is the diversity factor. The larger the *q* is, the more obvious the diversity of the wind speed is in space. *L* is the number of variable categories and *h* = 1, 2 … *L* is a specific type.$\;N_{h}$ and *N* respectively represent the number of units in *h* category and the number of units in the whole region. $\sigma_{h}$ and *σ* are the variance of h category and the variance of the whole region.

The mean annual wind speed and the maximum annual wind speed from 2001 to 2020 were regarded as the dependent variable Y1 and Y2, respectively. Potential driving factors, including Digital Elevation Model (DEM; X1), slope gradient (X2), slope aspect (X3), geomorphology type (X4), normalized difference vegetation index (NDVI; X5), air pressure (X6), temperature (X7), precipitation (X8), and land use type (X9) (Table 1) were selected as the detection factors X.

#### 2.4.4. Degree Classification of the Wind Impacts

According to the spatial distribution of the maximum annual wind speed in Spring and the strong wind days in water area of the typical black soil region, the wind impacts were classified into four categories using the natural breaks classification of GIS (10.2) proposed by George Frederick Jenks [41,42,43]. The specific steps are as follows: (i) classification of the strong wind days into four degrees as ‘mild, moderate, severe, extremely severe’; (ii) classification of the maximum wind speed in spring into four degrees as ‘mild, moderate, severe, extremely severe’; (iii) determining the degrees of the wind impacts as rules listed in Table 2.

## 3. Results and Discussion

### 3.1. Temporal Variation of Wind Speed

#### 3.1.1. Interannual Variation

**Figure 3:**
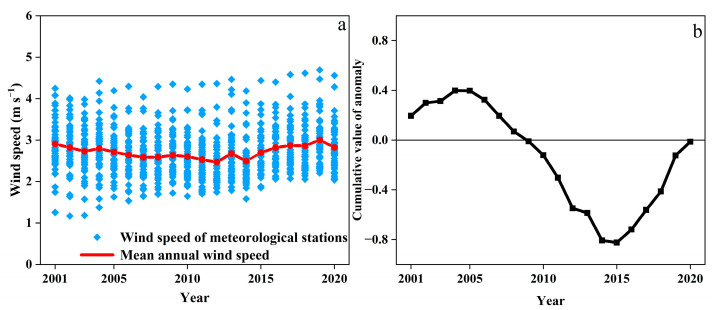
Interannual variation of the mean annual wind speed and its cumulative value of anomaly in water erosion area of the typical black soil region from 2001 to 2020. ((**a**): mean annual wind speed, (**b**):cumulative anomalous values of mean annual wind speed).

The mean annual wind speed ranged at 2.44–2.98 m s^−1^ during the years of 2001–2020, and averaged at 2.69 ± 0.14 m s^−1^ with the coefficient of variation (Cv) at 5.32% (Figure 3a). As shown in Figure 3b, the cumulative anomaly value of mean annual wind speed showed “S” type variations. The anomaly value of mean annual wind speed showed an upward trend during years of 2001–2005, decreased dramatically during the years of 2006–2014, and then increased in years of 2015–2020. The mean annual wind speed from 2001 to 2005 and 2015 to 2020 was larger than the average, while the mean annual wind speed in 2006–2016 was smaller than the average. In 2012, 2014, and 2019, the cumulative anomaly value changed significantly, and the mean annual wind speed changed abruptly during this period. Combined with Figure 3a,b, the variations of the mean annual wind speed could be classified into two stages as 2001–2014 and 2015–2020. From 2001 to 2014, the mean annual wind speed showed a downward trend with a decreasing rate of −0.0257 m s^−1^ a^−1^. From 2015 to 2020, the mean annual wind speed showed an upward trend with a rate of 0.0307 m s^−1^ a^−1^, and the increasing rate in recent years was greater than the decreasing rate in the past. Changes in wind speed are mainly affected by atmospheric circulation, monsoons, land use, and global warming caused by increasing human emissions of greenhouse gases [44]. The interannual variation of the mean annual wind speed was mainly affected by the abnormal cyclonic circulation in Northeast China [45]. In the year when cyclonic circulation is strengthened, the northwest wind anomaly occurs in the study area, which superimposed with the prevailing westerly wind in Spring, resulting in the increase of wind speed [46].

**Figure 4:**
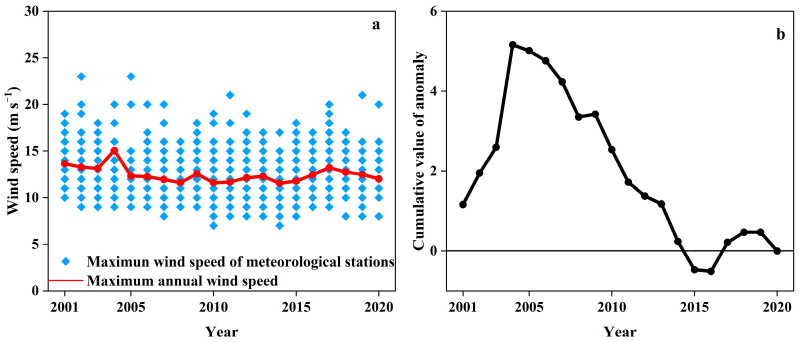
Interannual variation of the maximum annual wind speed and its cumulative value of anomaly in the water erosion area of the typical black soil region from 2001 to 2020. ((**a**): maximumu annual wind speed, (**b**):cumulative anomalous values of maximum annual wind speed).

From 2001 to 2020, the maximum annual wind speed showed a decreasing trend of −0.584 m s^−1^ a^−1^ with great fluctuations in the water erosion area of the typical black soil region, ranged at 11.55 m s^−1^ (2014)–15.06 m s^−1^ (2005) and averaged at 12.49 ± 0.83 m s^−1^, with the coefficient of variation (Cv) at 6.68% (Figure 4a). The anomaly value of maximum annual wind speed showed an upward trend during years of 2001–2004, decreased dramatically during the years of 2005–2014, and then increased in the years of 2015–2020. The maximum annual wind speed during the years of 2001–2004 and 2016–2019 was larger than the average, while the maximum annual wind speed in 2005–2015 was smaller than the average. The cumulative anomaly value changed significantly in 2004, 2014, and 2017. The maximum annual wind speed increased suddenly in 2004 while decreasing significantly in 2014. Generally, it showed a decreasing trend before 2014, an increasing trend during years of 2014–2017, and a decreasing trend during the years of 2018–2020.

**Figure 5:**
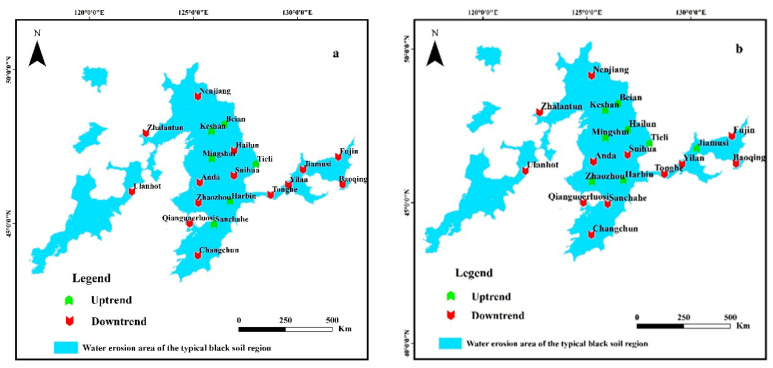
Mann–Kendall trend test of wind speed in water erosion area of the typical black soil region from 2001 to 2020 ((**a**): mean annual wind speed, (**b**): maximum annual wind speed).

Of the selected 51 meteorological stations, there were 20 meteorological stations in the water erosion area of the typical black soil region as Figure 5. According to the results of Mann–Kendall test, the changing trends of the mean annual wind speed and maximum annual wind speed around these 20 meteorological stations are shown in Table 3. In the 20 meteorological stations, the mean annual wind speed showed an upward trend in 6 stations and a downward trend in 14 stations, accounting for 30% and 70%, respectively. The maximum annual wind speed showed an upward trend in 8 stations and demonstrated a downward trend in 12 stations, accounting for 40% and 60%, respectively.

#### 3.1.2. Seasonal Variation within the Year

**Figure 6:**
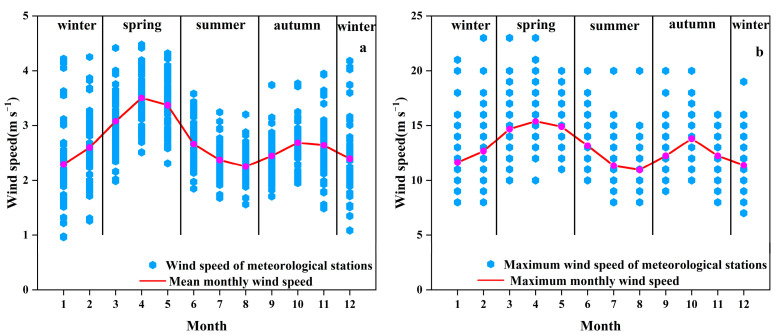
Seasonal variation of wind speed in water erosion area of the typical black soil region. ((**a**): mean monthly wind speed, (**b**): maximum monthly wind speed).

As shown in Figure 6, the mean monthly wind speed ranged from 2.5 to 4.5 m s^−1^, with an average of 3.4 m s^−1^. The maximum monthly wind speed ranged from 10.0 to 23.0 m s^−1^, with an average of 15.4 m s^−1^. Both the mean monthly wind speed and the maximum monthly wind speed in water erosion area of the typical black soil showed a “bimodal” trend within the year, and the high values were presented in April (mean: 3.50 m s^−1^; maximum: 15.39 m s^−1^) and October (mean: 2.69 m s^−1^; maximum: 13.80 m s^−1^), the highest being in April. In terms of the seasonal variation, they followed the order of Spring > Autumn > Winter > Summer. In Spring, the temperature rises rapidly, the pressure gradient difference changes, and the monsoon effect is strong, resulting in higher wind speed [46,47]. The vegetation grows vigorously in Summer, which plays a role in slowing down the wind. In addition, the temperature in Summer is generally high, and the low temperature difference makes the pressure gradient smaller to lower the wind speed as well. Autumn is the transition period between Summer and Winter, in which season the wind speed is higher due to the Mongolian high pressure [44,48]. In Winter, the temperature is low and the vegetation coverage is lower due to the harvest, which makes the overall wind speed in Winter higher than that in Summer [45,49].

### 3.2. Spatial Variation of Wind Speed

#### 3.2.1. Spatial Variation Characteristics of Wind Speed

**Figure 7:**
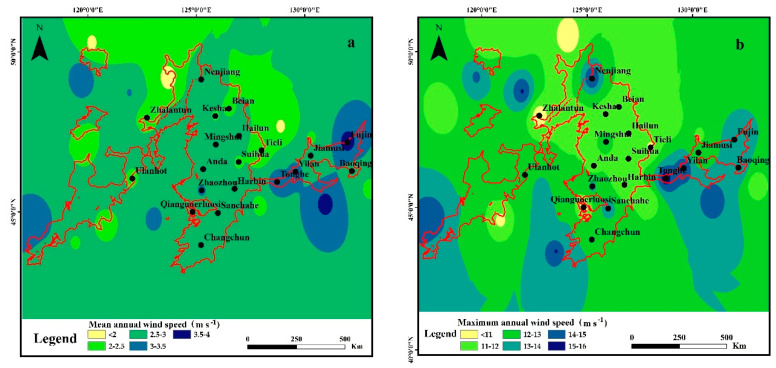
Spatial distribution of the wind speed in the water erosion area of the typical black soil region ((**a**): mean annual wind speed, (**b**): maximum annual wind speed).

As shown in Figure 7, the mean annual wind speed was generally high in the East and low in the northwest and northeast of the water erosion area in the typical black soil region. Specifically, the mean annual wind speed around Fujin, Yilan, and Tonghe meteorological stations in the East was the largest, with a variation range of 3–3.5 m s^−1^. The mean annual wind speed around Bei’an meteorological station in the middle was the smallest, with a variation range of 2–2.5 m s^−1^. The mean annual wind speed in the middle and west was between 2.5–3 m s^−1^.

The maximum annual wind speed in the water erosion area of the typical black soil region showed the decreasing trend as east > west > middle in general. The maximum annual wind speed in the East showed the highest value (15–16 m s^−1^) around Yilan and Tonghe meteorological stations, which was consistent with the highest value of the mean annual wind speed. The maximum annual wind speed in the west ranged at 12–13 m s^−1^ without great variations (16.6%). Compared with the east and west of the study area, the maximum annual wind speed showed higher spatial variation (50.4%) in the middle region, with a trend of first decreasing and then increasing from south to north. The east of the study area was located at the junction of Lesser Khingan Mountains and Changbai Mountains, where the higher near-surface wind speed is mainly due to the narrow tube effects in the local wind zone [44]. Affected by the altitude and terrain factors, the air flow is blocked and the maximum wind speed fluctuation is not obvious in the west of the study area, which is located in the Greater Khingan Mountains [46]. Compared with Figure 5 and Figure 7, it was noticeable that the wind speed in the middle of the study area is relatively small, but the change of both mean annual wind speed and maximum annual wind speed here showed upward trends. The influence of the wind speed in the middle of the study area should be concerned in the future.

#### 3.2.2. Spatial Variation of Wind Speed in April and May

**Figure 8:**
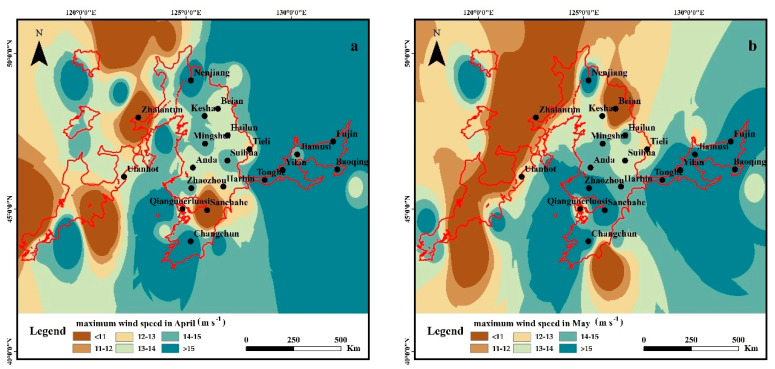
Spatial distribution of the maximum wind speed variation in April and May ((**a**): April, (**b**): May).

The spatial variation of the maximum monthly wind speed was specifically investigated in April and May (Figure 8), as it showed the highest value in these two months according to its seasonal variation characteristics in the study area. The maximum wind speed in April and May was higher in the east, southern, and northern sites of the study area, with a decreasing trend from east to west and south to north on the whole. There are three high values of the maximum speed (Figure 8a) in April, as: (i) the area around Yilan and Tonghe meteorological station in the east; (ii) the area around Nenjiang and Changchun meteorological station in the southern and northern sites of the middle; (iii) the area around Linxi meteorological station in the southern side of the west region. As shown in Figure 8b, compared with the maximum wind speed distribution in April, the maximum wind speed in May did not change greatly in the east, but significantly decreased in the area around Beian meteorological station in the north site of the middle study area, and increased significantly around Sanchahe station in the south side. Moreover, the wind speed decreased obviously in the west of the study area.

#### 3.2.3. Spatial Characteristics of Strong Wind Days

**Figure 9:**
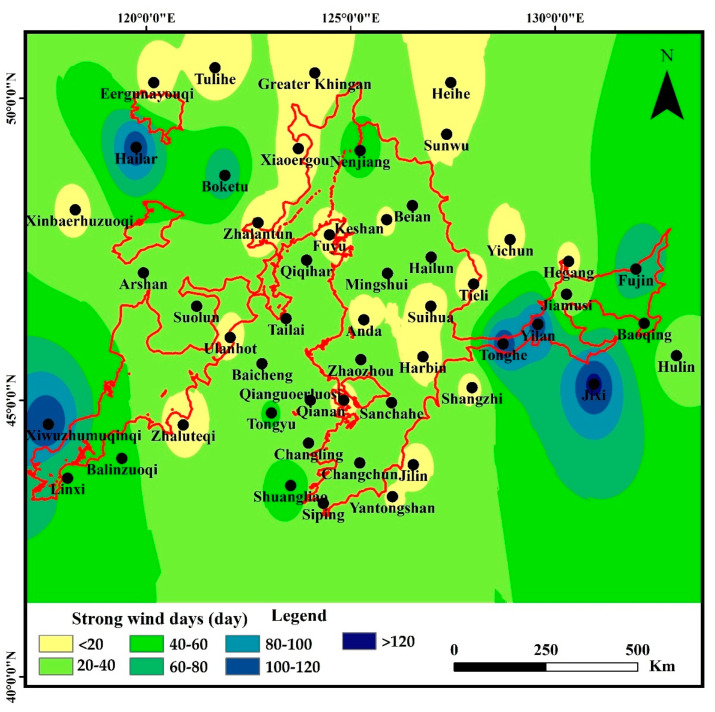
Spatial distribution of strong wind days in the water erosion area of the typical black soil region.

The strong wind days ranged at 4–127 days, averaged at 35 ± 32 days, and showed high heterogeneity in the water erosion area of the typical black soil region (Figure 9). Still, the strong wind days showed highest values in the east of the study area around Yilan stations (more than 120 days), similar to the Tonghe meteorological station. The strong wind days ranged at 4–60 days in the middle, with lower value (less than 20 days) in the central part of the middle around Tieli, Suihua, Anda, and Harbin stations. The strong wind days in the west of the study area ranged at 4–75 days, with the lower value around the Ulanhot stations. Wind is the most direct driving force of soil erosion. The greater the wind speed is, the stronger the wind erosion ability would be. Here, the strong wind means the situation of wind speed exceeds the threshold wind speed of sand movement (8 m s^−1^), thus the longer the strong wind days lead to a higher wind erosion capacity [50,51].

#### 3.2.4. Influencing Factors on Spatial Heterogeneity of Wind Speed

The analyzing results of the geographic detectors (Table 4) showed the contribution of each influencing factor on the spatial heterogeneity of the mean annual wind speed and the maximum annual wind speed. The effects of the selected influencing factors were not significant to the spatial variation of the man annual wind speed, as *p* values are much higher (1.000), despite the order of the influencing factor temperature (X7), precipitation (X8), DEM (X1), slope (X2), air pressure (X6), geomorphology type (X4), slope aspect (X3), and land use type (X9). DEM (X1) and temperature (X7) are the driving factors for the spatial heterogeneity of the maximum annual wind speed, as *p* < 0.005 and q > 0.08. The other factors have no significant impacts on the spatial variation of the maximum annual wind speed, with higher *p* values (1.000). The results are consistent with previous studies. For example, DEM was reported to have significant impacts on the wind speed, which was found to change the rate of air flow and change the horizontal and vertical shear of wind speed [52,53,54]. The effects of the temperature on the spatial variation of the wind speed are mainly due to the alteration of the pressure gradient by the temperature differences [55,56,57].

### 3.3. Wind Impacts Degree

**Figure 10:**
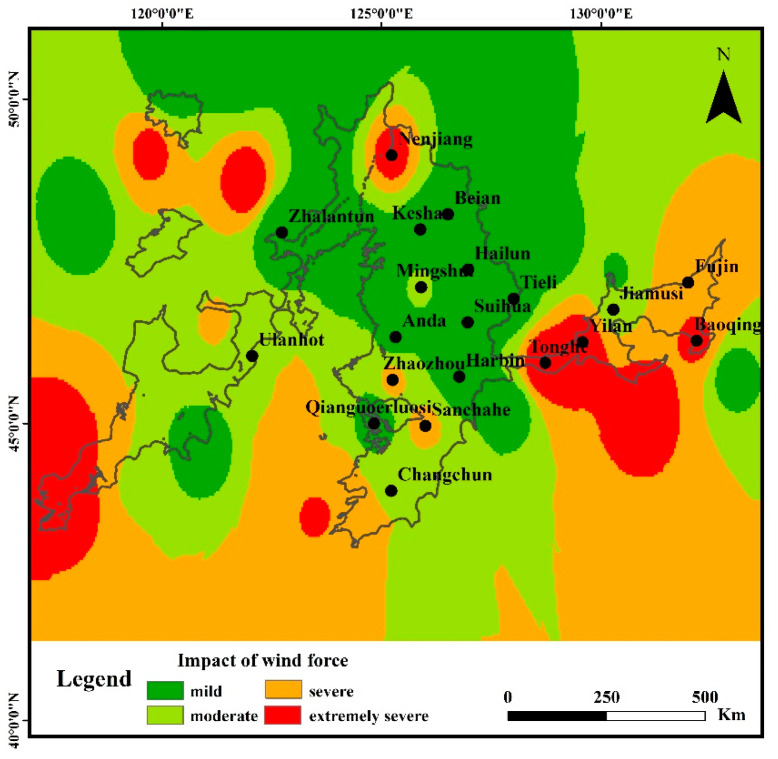
The degree of wind impacts in water erosion area of the typical black soil region.

The area distribution of the four degrees of wind impacts (mild, moderate, severe, extremely severe) are shown in Figure 10. The area with ‘mild’ degree of wind impacts dominated the study area, accounting for 40.2%, concentrated in the middle of the study area. The area with ‘moderate’ degree of wind impacts accounting for 36.4%, concentrated in the south part of the study area, especially around Ulanhot station in the west, Changchun in the south of the middle, Mingshui station in the north of the middle, and Jiamusi station in the east. The area with ‘severe’ degree of wind impacts accounting for 14.9%, mainly distributed in the east and west sides of the study area, scattered around Zhaozhou and Sanchahe station in the south of the middle. The area with ‘extremely severe’ degree of wind impacts accounted for 8.5%, and was distributed around Yilan, Tonghe, and Baoqing stations in the east, Nenjiang station in the north of the middle, and Linxi station in the west.

The land use type in the area of ‘severe’ and ‘extremely severe’ degree of wind impacts was cropland. Previous studies have shown a significant correlation between wind speed and land cover [44,58,59]. In Spring, the ground snow melts completely, and the cropland vegetation is sparsely covered. Wagner et al. indicated the positive correlations between the wind erosion amount and wind speed [49]. The exposed cropland without crops is more vulnerable to wind erosion in Spring, due to the rising temperature combined with snow melting and the highest wind speed in the year. The evaporation of surface water is strengthened due to the rise of temperature, and the rainfall is less in this period, which reduces the surface water content of cropland and makes it easier to be eroded by wind. Moreover, soil structure, surface roughness, and surface morphology could be changed under the action of wind in Spring, which would further impact the water erosion processes in Summer [28,29]. For example, previous studies indicated that the soil shear strength and soil hardness could be reduced by 2.9–8.2% and 4.4–12.3% by wind erosion, respectively, which aggravates water erosion [15,28,29]. Li et al. indicated that the wind erosion could lead to the coursing of the soil surface texture and increase the surface roughness by forming the wind erosion dent, which also affects the further water erosion [60]. The changing of the surface micro-morphology, like dents by the wind erosion, was also found to change the runoff path, increasing the runoff connectivity, runoff amount, and flow velocity in the subsequent water erosion [29]. The results of these previous studies are close to our results, which fully proves the influence of wind on water erosion, but its principle and effects need further study.

## 4. Conclusions

In this study, the spatial and temporal variation characteristics of wind speed from 2001 to 2020 were analyzed by the cumulative anomaly method, Mann–Kendall test method, and Kriging interpolation method. The main influencing factors of wind speed change were analyzed by geographic detectors method, and the influence of wind speed was classified. The maximum annual wind speed in water erosion area of the typical black soil region showed a downward trend, and the mean annual wind speed showed a downward trend first and then an upward trend. Under the background of global warming, atmospheric circulation is the main factor causing the temporal wind speed variation. The selected driving factors had no significant influences on the spatial variation of the mean annual wind speed, while DEM and temperature are the main factors affecting the spatial heterogeneity of the maximum annual wind speed. The impact of wind force in water erosion area of typical black soil region can be divided into four degrees: mild, moderate, severe, and extremely severe. Most of the study area (76.6%) was classified as a ‘mild and moderate’ degree of wind impacts. The rest of the study area was classified into the area with ‘severe’ and ‘extremely severe’ degree of wind impacts, where specific concern should be paid to the complex erosion processes by wind and water. Their impacts on grain output and food security should be further investigated for better understanding the complexing soil erosion processes in the typical black region. Wind speed is not only the basic parameter for determining the wind erosion, but also an important parameter for determining the wind impacts on the following water erosion processes. The method and results of this article could lay a basic foundation for the study of wind erosion impact, black soil resources protection, and high-efficiency utilization in black soil regions of the world. The coupling effects of complex erosion by wind and water and the coordination development of global black soil resources and agricultural sustainable development should be further investigated.

## Figures and Tables

**Table 1:** Driving factors for the Geographic detectors.

Classification	Detection Factor	Index
Terrain	X1	Digital Elevation Model (DEM)
	X2	Slope
	X3	Slope aspect
Geomorphology	X4	Geomorphological type (Plain, rolling hills, hill, alluvial terrace)
Vegetation	X5	Normalized Difference Vegetation Index (NDVI)
Climate	X6	Air pressure
	X7	Temperature
	X8	Precipitation
Land use	X9	Land use type (cropland, forest, grassland, shrubland, wetland, water body, artificial surface, bare land, tundra)

**Table 2:** Rules for degree classification of wind impacts.

Values of Different Degrees		Categories of the Impact of Wind Force
Maximum Wind Speed	Strong Wind Days	
mild	mild	mild
mild	moderate	mild
mild	severe	moderate
mild	extremely severe	severe
moderate	mild	moderate
moderate	moderate	moderate
moderate	severe	moderate
moderate	extremely severe	severe
severe	mild	moderate
severe	moderate	severe
severe	severe	severe
severe	extremely severe	severe
extremely severe	mild	severe
extremely severe	moderate	severe
extremely severe	severe	extremely severe
extremely severe	extremely severe	extremely severe

**Table 3:** Number of stations with different wind speed trends from 2001 to 2020.

Number	Name	Longitude	Latitude	Changing Trend	
				Mean Annul Wind Speed	Maximum Annual Wind Speed
1	Nenjiang	125.23	49.17	downtrend	downtrend
2	Zhalantun	122.73	48.00	downtrend	downtrend
3	Beian	126.51	48.28	uptrend	uptrend
4	Keshan	125.88	48.05	uptrend	uptrend
5	Hailun	126.97	47.43	downtrend	uptrend
6	Mingshui	125.90	47.16	uptrend	uptrend
7	Fujin	131.98	47.23	downtrend	downtrend
8	Ulanhot	122.05	46.08	downtrend	downtrend
9	Suihua	126.96	46.61	downtrend	downtrend
10	Anda	125.32	46.38	downtrend	downtrend
11	Tieli	128.01	46.98	uptrend	uptrend
12	Jiamusi	130.28	46.81	downtrend	uptrend
13	Yilan	129.58	46.30	downtrend	downtrend
14	Baoqing	132.18	46.32	downtrend	downtrend
15	Qianguoerluosi	124.83	45.00	downtrend	downtrend
16	Zhaozhou	125.25	45.70	downtrend	uptrend
17	Harbin	126.77	45.75	uptrend	uptrend
18	Tonghe	128.73	45.97	downtrend	downtrend
19	Sanchahe	126.00	44.96	uptrend	downtrend
20	Changchun	125.22	43.90	downtrend	downtrend
Summarized		Uptrend		6	8
		Downtrend		14	12
Pecentage		Uptrend		30%	40%
		Downtrend		70%	60%

**Table 4:** Analyzing results by the geographic factor tool.

Natural Factors	X1	X2	X3	X4	X5	X6	X7	X8	X9
q value (the mean annual wind speed)	0.085	0.054	0.008	0.009	0.012	0.031	0.176	0.110	0.007
*p*-value	1.000	1.000	1.000	1.000	1.000	1.000	1.000	1.000	1.000
q value (the maximum annual wind speed)	0.083	0.017	0.002	0.023	0.007	0.061	0.088	0.032	0.007
*p*-value	0.003	1.000	1.000	1.000	1.000	0.884	0.000	0.983	1.000

Note: X1 is digital elevation model (DEM), X2 is slope, X3 is slope aspect, X4 is geomorphological type, X5 is normalized difference vegetation index (NDVI), X6 is air pressure, X7 is temperature, X8 is precipitation, X9 is land use type.

## Data Availability

The datasets used and/or analyzed during the current study are avail-able from the corresponding author on reasonable request.
